# Impact of Leading by Example on Employees' Organizational and Job Psychological Ownership: A Moderated Mediation Study

**DOI:** 10.3389/fpsyg.2022.888653

**Published:** 2022-07-06

**Authors:** ZhiXiao Ye, Xianfa Shang, Zahid Shafait, Youli Xu

**Affiliations:** ^1^Department of Property Management, School of Management, Zhejiang Shuren University, Hangzhou, China; ^2^School of Economics and Management, Lanzhou University of Arts and Sciences, Lanzhou, China; ^3^School of Management, Northwestern Polytechnical University, Xi'an, China

**Keywords:** leading by example, organizational psychological ownership, job psychological ownership, organizational identification, leader-member exchange

## Abstract

This article studies the influence of leading by example on organizational psychological ownership and job psychological ownership. This article further introduces the mediating mechanism of organizational identification and the regulating mechanism of Leader–member Exchange (LMX). This study investigated 312 personnel from eight property management enterprises in East, Northwest, Northeast, and central China. This study adopts a quantitative research method, using survey data of project managers, team leaders, and managers of Property management projects in China. The data were collected by questionnaire survey. In terms of data analysis, AMOS 21.0 software was used to conduct structural equation modeling (SEM) using the maximum likelihood method to test direct and indirect effects. SPSS 25.0 software was used to test the moderating effect by multilevel regression analysis with the maximum variance method. Use these two methods to analyze the whole theoretical framework. The results established all assumed relationships. In this article, leading by example, one of the important dimensions of empowering leadership is studied as a new leadership style, and the predictive effect of leading by example on organizational psychological ownership and job psychological ownership is verified. This finding further verifies the influence mechanism and boundary conditions of empowering leadership in different dimensions. It is found that organizational identification has different mediating effects on leading by example and organizational psychological ownership and job psychological ownership. The moderating effect of LMX also further indicates that under the influence of Confucian pan-family culture, the leader's exemplary behavior with higher authority has a stronger influence on employees' organizational identification, organizational psychological ownership, and job psychological ownership. Their relationship is deeply influenced by the culture of China's unique organizational Circle Culture.

## Introduction

Since the 21st century, the development of Internet technology and the intensification of global competition have urged enterprises to replace the traditional hierarchical structure with a flat organizational structure (Jiang and Xu, 2020). A flat organizational structure deals with small centralized functions. The basic idea is that those who are responsible for implementing the decisions should also be the decision makers. Hence, the absence of middle managers places more authority cum responsibility, such as in decision making at the worker level (Andersson et al., 2019). To achieve a flat organizational structure, enterprises adopt authorization management which is a popular leadership type (Spreitzer, 1995). Empowering leadership and its implications in flat organizations has been the focal agenda of the theoretical circle and business arena (Cheng et al., 2021; Song and Chen, 2021). Leading by example as an empowering leadership style is an important dimension that influences followers through leading-by-example. Research has shown that followers respond strongly to the example set by a leader (Güth et al., 2007; Gächter et al., 2012). In this sense, a leader exhibits a set of behaviors that show commitment to their own work as well as the work of team members. This category includes behaviors such as working as hard as he/she can and working harder than team members (Arnold et al., 2000). Empowering leadership makes sure to disseminate individual cum organizational support for employees and this in turn, contributes strongly toward mutual bonds of leader–member and organizational success (Rasool et al., 2022). Once a positive functional organizational environment is created, it is maintained through repeated strong mutual relationships of leader–member hence corroborating long-term organizational success (Rasool et al., 2019).

According to the psychology of possession theory, employee psychological ownership helps to improve empowerment, and motivates employees to work and trust the organization, while organizational identification influences decision making, work attitude, motivation, job satisfaction, performance, and goal attainment within the organization (Cheney, 1983). Psychological ownership further, refers to an individual's sense of possession of the object (Pierce and Kostovat, 2001). Individuals have different psychological ownership of different objects, including organizational psychological ownership of the entire organization (Dyne and Pierce, 2004) and job psychological ownership directed at specific jobs (Pierce et al., 2004). Those entrusted with power and responsibilities by leaders are considered loyal; have strong psychological belonging and attachment to the organization (O'Reilly and Chatman, 1986; Ashforth and Mael, 1989). Enterprises intend to improve the organizational psychological ownership (such as employee stock ownership plan) and job psychological ownership (such as enhancing the meaning of work) to stimulate employees' work initiative and self-management consciousness, to improve employee satisfaction, attendance rate, and work performance (Rhodes and Steers, 1981). This trust further motivates employees to perform better while encouraging them to avoid occupational stress (Rasool Samma et al., 2020). If organizations lack trust in a leader–member relationship, then it may have a severe impact on employees' engagement and their wellbeing; moreover, organizational support may be then nullified if the mutual combination of leader–member has no care and respect (Rasool et al., 2021).

Organizational identification explains the psychological connection and mechanism between employees and organizations further and predicts employees' positive work attitudes and behavior (Jian et al., 2017). Organizational identification is a special form of Social Identity Theory (SIT) that refers to a sense of belonging and loyalty to an organization. Previous studies have shown that organizational identification has a mediating effect in many contexts (He and Ling, 2008; Li and Xu, 2014; Shi et al., 2015; Cao et al., 2019), but a few existing literature studies focus on the role of organizational identification—mediating mechanism between leading by example and organizational psychological ownership and job psychological ownership.

According to the interactive determinism of Bandura (1977), “behavior, cognition, and environment” are interconnected and mutually determined in the process of social learning. Therefore, leading by example has an impact on employees' attitudes and behaviors, and will also be interfered and influenced by the environment, such as Leader–Member Exchange relationships (LMX) (Wang, 2013). Positive work anatomy helps employees to incorporate a positive working environment hence circumventing a toxic workplace environment and diminished worker productivity (Rasool et al., 2019). Due to the limited time and energy of leaders, they adopt different management styles for subordinates with different intimate relationships in work hence, different subordinates are divided into “in-Group member” and “out-Group member” by leaders. Among them, “in-group member” gets more trust and care from leaders, and even enjoys certain privileges, such as more autonomy, voice, promotion opportunities, and remuneration. The other subordinates become “out-group member,” whose relationship with the leader is limited to the scope of a formal working relationship, contact with the leader is limited to working hours, and salary, reward, and promotion are limited to normal work scope (Graen and Uhl-Bien, 1995). This phenomenon of “in-Group member” and “out-Group member” exists in organizational settings (Green et al., 1996). It should be interesting to see the moderating effect of the greater contrast between the two.

With the substantial increase in labor costs in China, the low-cost strategic advantage of Chinese enterprises in the global competition is gradually weakening. This, in turn, forces Chinese enterprises to establish effective organizational empowering leadership that matches the flat organizational structure to form a good authorization environment, which enables organizations to obtain sustained competitive advantages (Quinn and Keough, 2002). Therefore, this article tries to explain that the positive influence of leading by example is mainly directed at the psychological ownership of the organization or work, to improve the independent decision-making ability and empowerment ability of middle and lower-level management cadres and employees in enterprises. Taking middle-level cadres and managers of property management enterprises in China as samples, this article discusses the influence of leadership style on employees' attitude and behavior boundary conditions through intermediary mechanisms. LMX variables are introduced in this article to test the moderating effect of LMX on the relationship between leading by example and organizational identification, organizational psychological ownership, and job psychological ownership, and further clarify the boundary conditions and application scope of the research model.

Based on the self-determination theory(SDT)with the path of “leadership style—work motivation—impact,” this study intends to investigate the following research questions:

RQ1: How does leading by example influence employees' organizational and job psychological ownership?
RQ2: How does organizational identification mediate between leading by example and employees' organizational and job psychological ownership?
RQ3: How does LMX moderate between leading by example and organizational identification?
RQ4: How does LMX moderate between employees'organizational and job psychological ownership?

The article is structured as follows: Section **Theory and Hypotheses** is devoted to the literature review, hypotheses development, and explanation of the research model. Section Research Methodology shows the research methods of the study in detail, and section Data Analysis and Research Results presents the analysis, results, and their interpretation. Section Discussion provides a comprehensive discussion, and section **Practical Implications, Limitations, and Future Research** presents the practical implications, limitations, and future research directions concluded through this study. The section Conclusion presents the conclusions of the study.

## Theory and Hypotheses

### Leading by Example, Organizational Psychological Ownership, and Job Psychological Ownership

Empowering leadership (leading by example is a sub-dimension of empowering leadership (Arnold et al., 2000) is a leadership style that emphasizes empowering employees to work autonomy and stimulating employees' internal motivation and self-efficacy (Carmeli et al., 2011). Leaders share power with employees by clarifying the meaning of work, providing work autonomy, expressing trust in employees' abilities, and encouraging employees to participate in decision making (Ahearne et al., 2005).

Since Manz et al. (2001) put forward the concept of empowering leadership in the 1990s, the academic circle has studied the influence of empowered leadership on individuals, groups, and organizations from the perspective of “relationship” and “employee motivation,” respectively. Existing studies have shown that, at the individual level, empowering leadership has a significant impact on employees' psychological empowerment, organizational commitment, job satisfaction, and innovation behavior (Lin and Ling, 2016). At the group or team level, empowering leadership has a significant impact on knowledge sharing, team learning, team creativity, and team performance (Pearce, 2002; Zhang and Bartol, 2010; Xuey and Liang, 2011). At the organizational level, empowering leadership has a positive impact on enterprise performance and subordinate behavior. It can be seen that empowering leadership can significantly improve subordinates' attitudes and behavior toward work or organization. Leadership styles including empowering leadership has a significant positive impact on psychological ownership (Avey et al., 2009; Chen, 2010; Li et al., 2018).

Thus, it is certain that leading by example is a vital dimension of empowering leadership hence, the corresponding influence mechanism plays a role. Therefore, leading by example can help employees acquire a sense of belonging, self-efficacy, and self-identity in the organization and work. Consequently, improves employees' organizational psychological ownership and job psychological ownership. Thus, the following hypotheses are established from the mentioned arguments:

**Hypothesis H1a:** leading by example has a significant positive impact on organizational psychological ownership.
**Hypothesis H1b:** leading by example has a significant positive impact on job psychological ownership.

### Leading by Example and Organizational Identification

Based on social identity theory (SIT), organizational identification is a state that defines the self as an organization member or a perception of belonging to a group, which is a specific form of social identity (Ashforth and Mael, 1989). Organizational identification, therefore, is a self-construct derived from the employee as a member of the organization and will produce values and emotional connections to conform to the organization (Tajfel et al., 1970). In the context of China, the exemplary leadership of business leaders is a highly valued means of management and motivation. Based on the social learning theory **(SLT)**, leaders should not only set an example, but also commend and reward model employees and set up learning examples to show all employees the behaviors advocated by the organization. These behaviors are easy to be recognized and supported by employees.

In view of the leader's status and authority, the direct information or subconscious emotional signals conveyed by the leader's behavior will profoundly affect the subordinates' self-concept cognition. In an organization, a good relationship between superiors and subordinates will make employees feel warm, thus improving their sense of existence and belonging in the organization. This process will enable employees to record feedback on their work with a positive attitude, showing a higher sense of work involvement with self-efficacy, and expecting to realize their self-value more quickly. Some studies have found a significant positive correlation between certain leadership styles including empowering leadership and organizational identification (Li and Shi, 2005; Pellegrini and Scandura, 2006; Chen, 2010). Leadership behavior often represents the image of the organization (Konczak, 2000). Leaders convey organizational values and power distribution principles through exemplary behavior, thus exerting a direct influence on subordinates' attitudes and behaviors. Therefore, we predict that leading by example will have an impact on subordinates' organizational identification, putting forward the following hypothesis:

**Hypothesis H2:** Leading by example has a significant positive impact on organizational identification.

### Organizational Identification, Organizational Psychological Ownership, and Job Psychological Ownership

Organizational identification, originated from social identity, is a core concept of organizational research. Organizational identification is the process of forming the consistency between individuals and organizational goals and values while taking organizational values and norms as part of self-concept (Ashforth and Mael, 1989; Ashforth et al., 2008). Organizational identification reflects the potential connection between individuals and organizations, can affect individuals' psychology, and then change their work attitude and behavior. Organizational identification can promote employees to have positive work attitudes and behaviors (Riketta, 2005). The positive effects of organizational identification, such as job satisfaction, cooperative intention, job engagement, the subjective wellbeing of job performance, and reduction of employee stress and burnout have been confirmed and generally accepted by scholars (Wu, 2012; Avanzi et al., 2015; Kirstien et al., 2015; Yang et al., 2015).

Employees with high organizational identification regard the interests of the organization as the primary consideration, and even without external supervision, they can take the initiative to show behaviors in line with the interests of the organization (Albert et al., 2000). Organizational identification influences decision making, work attitudes, motivation, job satisfaction, and job performance within the organization (Cheney, 1983). Employees with high organizational identification have a stronger sense of trust and belonging to the organization, which affects the psychological ownership of the organization and is more likely to make decisions in line with the interests of the organization (Bao and Xu, 2006; Zhang et al., 2014). Studies have shown that organizational identification has a significant impact on organizational citizenship behavior (Dukerich et al., 2002), job satisfaction (Dick et al., 2004), turnover intention, increase employee participation (Riketta, 2005), and work attitude and performance (Mignonac et al., 2006). Employees with high organizational identification will regard organizational goals as personal goals, prompting them to work harder and act in accordance with organizational values and norms to achieve these goals, thus affecting employees' job psychological ownership.

Since organizational identification can certainly improve employees' organizational psychological ownership and job psychological ownership, we put forward the following hypotheses:

**Hypothesis H3a:** Organizational identification has a significant positive impact on organizational psychological ownership.
**Hypothesis H3b:** Organizational identification has a significant positive impact on job psychological ownership.

### The Mediating Role of Organizational Identification

Research on antecedents of organizational identification and leadership styles is considered a vital influencing factor. For example, humble leadership and transformational leadership have been proved to positively predict organizational identification (Qu et al., 2013; Wang and Zhuo, 2014). Furthermore, organizational identification comes from the sense of belonging and identity given to employees by leaders (Mael and Ashforth, 1992). Organizational identification has been proved to have a good mediating effect in various contexts in many studies (Li and Xu, 2014; Cao et al., 2019; Qiu et al., 2019). Previous studies have shown that organizational identification plays a mediating role between individual self-concept and employee organizational behavior (Li and Xu, 2014; Shi et al., 2015).

Followers' trust in leaders is positively correlated with organizational identification. Employees with high organizational identification tend to regard organizational goals as their personal goals, prompting them to work harder and act in accordance with the values and norms of the organization to achieve organizational goals. Li et al. (2018) found that empowering leadership can help employees acquire a sense of belonging, self-efficacy, and self-identity in their organization and work, thus improving their organizational psychological ownership and job psychological ownership. It can be, therefore, argued that leading by example can affect individual psychology through organizational identification, enhances employees' psychological belonging, changes their work attitude and behavior, and promotes organizational psychological ownership and job psychological ownership, hence, we put forward the following hypotheses:

**Hypothesis H4a:** Organizational identification plays a mediating role between leading by example and organizational psychological ownership.
**Hypothesis H4b:** Organizational identification plays a mediating role between leading by example and job psychological ownership.

### The Moderating Effect of LMX

Leader–member exchange theory was developed from early vertical dual links (Graen and Uhl-Bien, 1995). Leader–member exchange theory (LMX) has become a very important theory in the study of leadership behavior (Miner, 2002). Due to the limited time and energy of managers themselves, they cannot allocate resources equally to each subordinate, so they will establish exchange relations of different quality with subordinate employees. Hence, employees get resources or rewards from their leaders, such as trust and respect, salary increases and promotion opportunities. LMX includes four dimensions, namely, emotion, contribution, loyalty, and professional respect (Dienesch and Liden, 1986; Liden and Maslyn, 1998). Emotion refers to the emotional experience between leaders and subordinates based on mutual personal attraction rather than work or professional knowledge. Mutual attraction is the core factor that affects the exchange quality between leaders and members. Many studies have shown that emotion is a key dimension of the leader–member exchange relationship (Wayne and Ferris, 1990). The emotions of leaders and members are the basic factors affecting trust, loyalty, and responsibility. Loyalty refers to the public support of one leader or member for the conduct and character of the other. Loyalty is the foundation of trust. Contribution refers to the perception of the quantity, direction, and quality of the efforts made by the leading members of the exchange relationship toward the common goal. Professional respect refers to the degree to which leaders and members have a perception of each other's reputation in the workplace. A high degree of professionalism on the job is a core factor in developing high-quality relationships.

Furthermore, the proportion of preferences in different dimensions may be different, thus affecting the quality of exchange relations between leading members, and its moderating effects on work or organization may be different (Dienesch and Liden, 1986; Bridge and Baxter, 1992). Yao and Le (2011) found differences in the regulatory action modes of LMX in different dimensions when studying the relationship between LMX regulating expectation gap and adaptation. Therefore, the quality of the exchange relationship between leading members has a moderating effect in different situations (Dienesch and Liden, 1986). For example, LMX has a positive moderating effect on organizational trust and knowledge sharing (Zhao et al., 2010). LMX plays a moderating role in the relationship between authentic leadership and employee innovation behavior (Han and Yang, 2011).

The most distinctive feature of Chinese culture is the emphasis on interpersonal relationships. In Chinese organizations, the interaction between superiors and subordinates is the key to the operation of an organization (Tjosvold, 1985). Leader–member LMX is at the core of relationships within an organization. The quality of leader–member exchange relationship determines the effectiveness of leadership and organizational performance. For example, the quality of leader–member exchange directly affects subordinates' job performance, out-of-role behavior, job involvement, job satisfaction, and turnover intention (Graen and Uhl-Bien, 1995). It can be seen that the quality of leader–member exchange relationship may have different effects on organizational identification, organizational psychological ownership, and job psychological ownership.

Therefore, the interaction between the quality of leader–member exchange relationship (LMX) (Dienesch and Liden, 1986) and leading by example will have different effects on organizational identification, organizational psychological ownership, and work psychological ownership. In other words, compared with the low LMX relationship, the high-quality LMX relationship can enhance the strength of the positive relationship between leading by example on organizational identification, organizational psychological ownership, and job psychological ownership. We predict that LMX plays a moderating role in the relationship between leading by example and organizational identification, organizational psychological ownership, and job psychological ownership, therefore, we put forward the following hypotheses:

**Hypothesis H5a:** LMX moderates the relationship between leading by example and organizational psychological ownership.
**Hypothesis H5a:** LMX moderates the relationship between leading by example and job psychological ownership.
**Hypothesis H6:** LMX moderates the relationship between leading by example and organizational identification.

### Hypothesized Research Model

**Figure 1:**
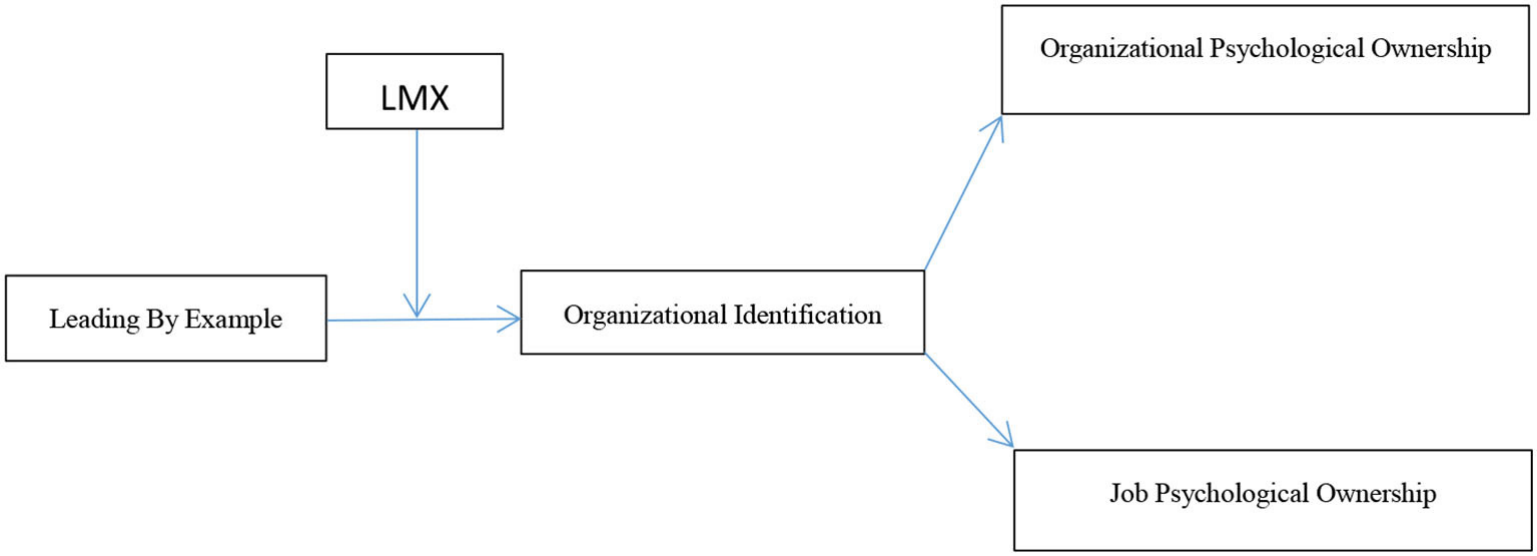
The hypothesized research model.

Based on the above assumptions, the following model is formed (as shown in Figure 1).

## Research Methodology

### Research Approach

This study adopts the method of a questionnaire survey to collect a wide range of samples from specific people in specific industries, which is relatively low cost (Heeringa et al., 2017; Rasool Samma et al., 2020; Shafait et al., 2021a). In this study, the maximum likelihood structural equation model was used to test the direct effect and indirect effect, and the maximum variance method was used to analyze the moderating effect, to analyze the theoretical framework. The data collection process utilized national property management cadres training conference and project managers from all over the country through questionnaires. In addition, the researchers also took advantage of the summer vacation travel to the Xinjiang Autonomous Region, Qinghai province, and other provinces and cities in the property service enterprises and conducted a questionnaire survey and interviews. Interviews and tests were conducted with enterprise personnel, and records were sorted out while the translations of questionnaire items were corrected. The property management project was regarded as a work team, and the leaders and employees were paired and sent questionnaires, respectively. A total of 375 questionnaires were sent out through convenience sampling (a time-and-cost effective means of data collection often used for social and business research) (Shafait et al., 2021b) and 312 valid questionnaires were recovered, lasting for 100 days.

### Questionnaire Development

Maturity scales such as Arnold et al. (2000) were mainly used in this study. Due to the low level of education of employees engaged in property management, in the process of questionnaire design, we not only solicited the evaluation of experts in the field of project management (Zaman et al., 2022) but also solicited the opinions of some employees, making the questionnaire more understandable and effective. Among them, there are five academic professors, four doctoral professors, 15 postgraduates, 22 property management project managers, and their subordinate employees. We found the four local property service companies, and the property management project managers and employees were paired for a pre-test. Interviews and tests were conducted on enterprise personnel, records were sorted out and evaluations filled in within 24 h, the translation of questionnaire items was corrected, and the scale was finally determined after the prediction of 46 questionnaires. The property management project was regarded as a work team, and the leaders and employees were paired and sent questionnaires, respectively. Eight property management enterprises from East China, northwest China, North China, and Northeast China were chosen for the study of grassroots managers. The subjects were distributed in Hangzhou, Zhejiang province, Urumqi, Xinjiang Province, Delingha, Qinghai Province, Suzhou, Jiangsu Province, Changsha, and Hunan Province.

### Measures

A 7-point Likert scale was used for data collection in which 1 = “strongly disagree” and 7 = “strongly agree.” Items comprising each scale were averaged to create composite measures for each variable.

#### Leading by Example

In this article, the “LEB Scale” developed by Arnold et al. (2000) is adopted. The breakdown of these items was LBE (5 items), Participative Decision Making (six items), Coaching (11 items), Informing (six items), and Showing Concern/Interacting with the Team (10 items). Since this article focuses on LBE factors, only LBE dimension is selected. The Cronbach's alpha for this scale was 0.938, and it has a very high-reliability coefficient.

#### Organizational Psychological Ownership and Job Psychological Ownership

In this article, job psychological ownership scale developed by Pierce et al. (2004) is adopted. Furthermore, organizational psychological ownership scale developed by Dyne and Pierce (2004) is adopted. It has two dimensions pointing to OPO and JPO, respectively, including “this is MY organization,” “I sense that this organization is our company,” and so on a total of 12 items. The Cronbach's alpha for OPO was **0.774**. The Cronbach's alpha for JPO was **0.87**.

#### Organizational Identification

Organizational identification (OI) scale with 6 items was adopted from Mael and Ashforth (1992), which includes “when someone criticizes my organization, it feels like a personal insult,” “I am very interested in what others think about my organization,” and so on a total of six items. The Cronbach's alpha for this scale was **0.869**.

#### Measurement and Questions for LMX

In this article, the LMX Scale developed by Liden and Maslyn (1998) is adopted. Dienesch and Liden (1986) believed that leader–member exchange was a multidimensional construct, namely, three dimensions of affect, contribution, and loyalty. Later, Liden and Maslyn (1998) added the dimension of professional respect as the fourth dimension of leader–member exchange, forming the current four-dimensional structure of LMX. The breakdown of these items was affect (three items), loyalty (three items), contribution (two items), and professional respect (three items). Items include “like my supervisor very much as a person.” and so on a total of 10 items. The Cronbach's alpha for this scale was **0.937**.

In addition, the main demographic variables of employees were treated as control variables, such as age, gender, length of service, and education.

## Data Analysis and Research Results

### Reliability and Validity

According to the descriptive analysis of each variable, the maximum kurtosis of the data of all items and the mean value of variables are 3.446 and 3.140, respectively, and the absolute value is <10. The maximum skewness value is 1.67 and −1.546, and its absolute value is <3, indicating that item data and variable mean data of all variables are in line with the normal distribution (Huang, 2005).

#### Results of Factor Loadings and Reliability Tests

As shown in Table 1. The total correlation CITC value of the corrected items is all higher than 0.5, indicating that the correlation between the items is high and the Cronbach's α coefficient of the items is high. According to the reliability test results of each scale, the Cronbach's α value above 0.7, the composite reliability (CR) ranged from 0.771 to 0.942, meeting the criteria of combined reliability above 0.7 (Fornell and Larcker, 1981), indicating that the questionnaire had good reliability.

**Table 1:** Results of factor loadings, reliability, and validity tests.

**Variable**	**Items**	**CITC**	**Estimate**	**Cronbach'α**	**C.R**.	**AVE**
LBE1	Sets high standards for performance by his/her own behavior	0.835	0.899	0.938	0.954	0.804
LBE2	Works as hard as he/she can	0.870	0.921			
LBE3	Works as hard as anyone in my work group	0.827	0.891			
LBE4	Sets a good example by the way he/she behaves	0.881	0.927			
LBE5	Leads by example	0.764	0.844			
OPO2	I feel a very high degree of personal ownership for this organization.	0.615	0.833	0.774	0.872	0.693
OPO3	Most of the people that work for this organization feel as though they own the company.	0.630	0.844			
OPO4	I sense that this is MY company.	0.598	0.821			
JPO10	I sense that this job is MY job and I took on it.	0.754	0.893	0.870	0.920	0.793
JPO11	I sense that this job is MY job and I take responsibility for it.	0.800	0.916			
JPO12	I sense that that what I do work is part of who I am.	0.702	0.862			
OI3	When I talk about companies, I say “we do” instead of “they do.”	0.656	0.831	0.869	0.921	0.796
OI4	The success of the company is also my success	0.811	0.927			
OI5	When people compliment the company I work for, I feel like I'm being complimented	0.790	0.916			
LMX1	like my supervisor very much as a person.	0.786	0.841	0.937	0.949	0.700
LMX2	My supervisor is the kind of person one would like to have as a friend.	0.794	0.847			
LMX3	My supervisor is a lot of fun to work with.	0.788	0.845			
LMX6	My supervisor would defend me to others in the organization if I made an honest mistake.	0.740	0.798			
LMX8	I am willing to apply extra efforts, beyond those normally required, to further the interests of my work group.	0.658	0.730			
LMX9	I am impressed with my supervisor's knowledge of his/her job.	0.849	0.892			
LMX10	I respect my supervisor's knowledge of and competence on the job.	0.811	0.863			
LMX11	I admire my supervisor's professional skills.	0.818	0.868			

*LBE, Leading By Example; OI, Organizational Identification; OPO, organizational psychological ownership; JPO, job psychological ownership; LMX, Leader-Member Exchange Relationships*.

Validity analysis mainly includes three parts: content validity, convergent validity, and discriminant validity. This article determines the appropriateness of content validity through expert research and logical reasoning. AVE values of Leading by Example, Organizational Psychological Ownership, Job Psychological Ownership, Organizational Identification, and Leader–Member Exchange (LMX) are 0.804, 0.693, 0.793, 0.796, and 0.7, respectively, and are all greater than the standard value of 0.5 (Fornell and Larcker, 1981).

### Descriptive Statistics, Correlation Coefficients, and Common Method Bias

The correlation coefficients (Table 2) of each factor are 0.477, 0.358, 0.460, 0.731, 0.483, 0.433, 0.578, 0.488, 0.468, and 0.551, respectively. The square root AVE values of the five factors were 0.897, 0.892, 0.832, 0.891, and 0.837, respectively, indicating that the root mean square AVE values of all factors were greater than the correlation coefficients between this fact and/or other factors. This indicates that our scale has good discriminant validity (Fornell and Larcker, 1981).

**Table 2:** Discriminatory validity analysis of each variable.

	**Mean**	**SE**	**LBE**	**OI**	**OPO**	**JPO**	**LMX**
Leading by example	6.15	0.055	(0.897)	0.477**	0.358**	0.460**	0.731*
Organization identification	6.03	0.055		(0.892)	0.483**	0.433**	0.578**
Organizational psychological ownership	5.53	0.067			(0.832)	0.488**	0.468**
Job psychological ownership	5.59	0.064				(0.891)	0.551**
Leader-member exchange	5.92	0.058					(0.837)

** < 0.01.

In order to determine the discriminant validity again, AMOS21.0 was used to conduct confirmatory factor analysis (CFA) for five variables, including LBE, LMX, OI, OPO, and JPO. The CFA fitting index of the five-factor model was the best as follows: χ^2^/df = 1.73; CFI = 0.979; IFI = 0.98; TLI = 0.974; NFI = 0.95; PGFI = 0.67; AGFI = 0.913; RMSEA = 0.049; SRMR = 0.036. And all indexes are better than other factor models, which again shows that the model has good validity. At the same time, it also shows that there is no common method bias (CMB).

### Testing of Research Hypotheses

**Figure 2:**
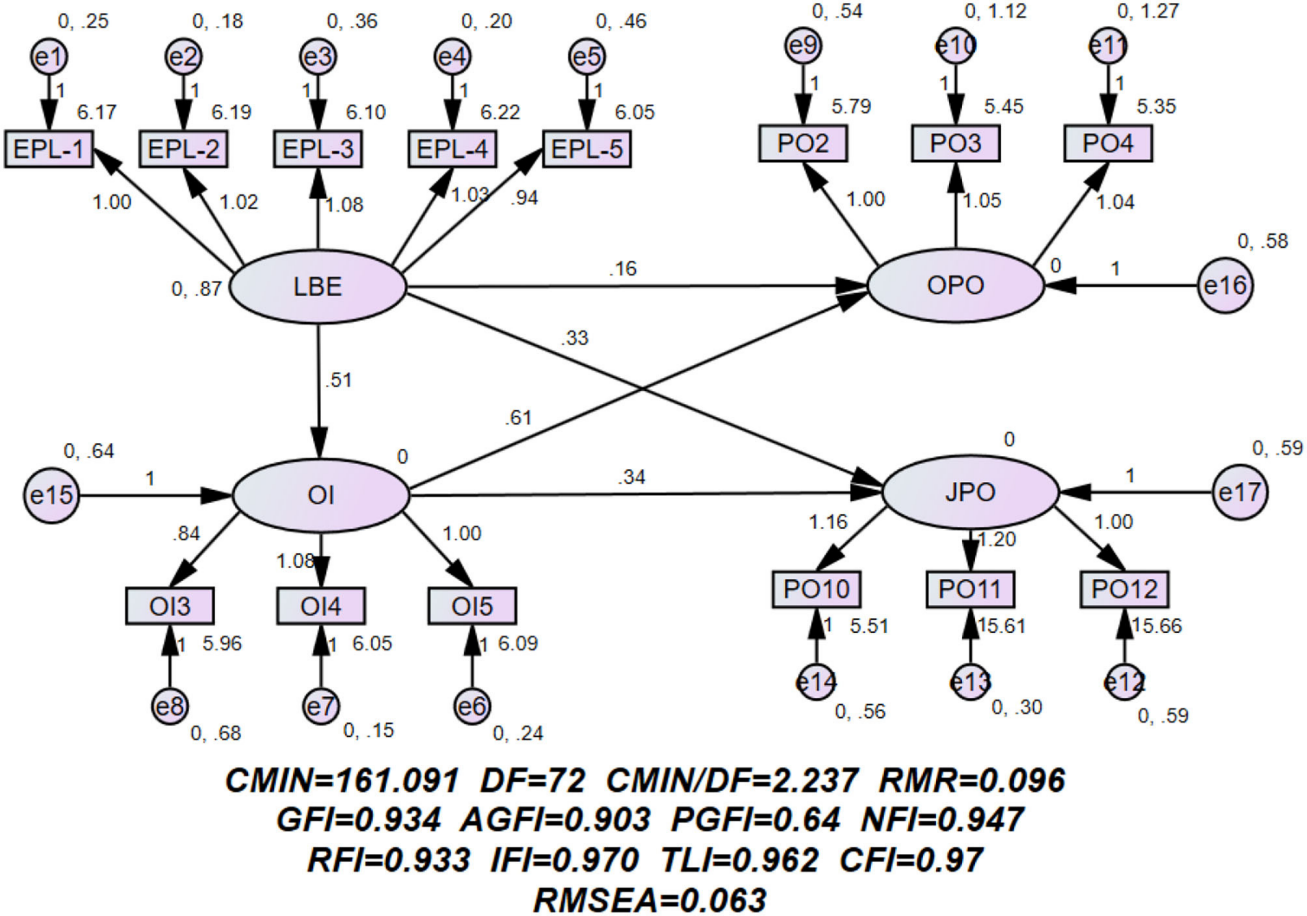
The mediation of organizational identification between leading by example and organizational psychological ownership and job psychological ownership.

An analysis of the mediating effect of organizational identification. This study employs structural equation modeling supported by AMOS 21.0 (Hair, 1998; Preacher and Hayes, 2008). The fitting indexes of the research model (see Figure 2, Table 3) is as follows: χ^2^/df = 2.237; GFI = 0.934; AGFI = 0.903; IFI = 0.970; TLI = 0.962; CFI = 0.97; NFI = 0.947; RMSEA = 0.063; SRMR = 0.059. Thus, it is proven that the model has a good fit and provides sufficient support for the results.

**Table 3:** Summary of model fit.

**Fit index**	**Ideal value**	**Model (LBE-OI-PO)**	
		**Result**	**Conclusion**
χ^2^/df	<3 (good fit) <5 (acceptable fit)	2.237	Good fit
GFI (Goodness of Fit Index)	>0.9 (good fit) 0.8–0.89 (acceptable fit)	0.934	Good fit
AGFI (Adjusted Goodness of Fit Index)	>0.9 (good fit) 0.8–0.89 (acceptable fit)	0.903	Good fit
NFI (Normed Chi-square Index)	>0.9 (good fit) 0.8–0.89 (acceptable fit)	0.947	Good fit
IFI (Incremental Fit Index)	>0.9 (good fit) 0.8–0.89 (acceptable fit)	0.970	Good fit
TLI (Tucker-Lewis Index)	>0.9 (good fit) 0.8–0.89 (acceptable fit)	0.962	Good fit
CFI (Comparative Fit Index)	>0.9 (good fit) 0.8–0.89 (acceptable fit)	0.97	Good fit
RMSEA (Root Mean Square Error of Approximation)	≤ 0.05 (close fit) 0.05–0.08 (fair fit) 0.08–0.10 (mediocre fit)	0.063	fair fit
SRMR (Standardized Root Mean Square Residual)	−1 < Standardized RMR <1 (good fit)	0.0592	Good fit

Structural equation path analysis method was adopted, and the results are shown in Table 4. The Bias-Corrected Bootstrap procedure was used to test the significance of the mediating effect of organizational identification, and the indirect effect results were tested with 2,000 Bootstrap tests (Preacher and Hayes, 2008), as shown in Table 5. The result follows:

**Table 4:** Summary of model fit.

**Model**	**Path**	**Estimate**	**S.E**.	**C.R**.	***P***
Model 1	LBE → OI	0.506	0.058	8.766	***
	LBE → OPO	0.332	0.066	5.027	***
	LBE → JPO	0.159	0.071	2.243	0.025
	OI → OPO	0.608	0.077	7.870	***
	OI → JPO	0.341	0.067	5.080	***

*** < 0.00, ** < 0.05, * < 0.1.

**Table 5:** Indirect effect test.

**Model path**		**Estimated effect**	**Sig**.	BC 95%CI*	
				**Lower bounds**	**Upper bounds**
Total effects	LBE → OI	0.508**	0.001	0.366	0.624
	LBE → OPO	0.433**	0.001	0.293	0.574
	LBE → JPO	0.499**	0.001	0.392	0.594
	OI → OPO	0.563**	0.001	0.421	0.705
	OI → JPO	0.336**	0.001	0.178	0.496
Direct effects	LBE → OI	0.508**	0.001	0.336	0.624
	LBE → OPO	**0.147**	0.064	**−** **0.008**	**0.310**
	LBE → JPO	0.328**	0.001	0.196	0.449
	OI → OPO	0.563**	0.001	0.421	0.705
	OI → JPO	0.336**	0.001	0.178	0.496
Indirect effects	LBE → OI → OPO (full mediation)	0.286***	0.000	0.199	0.410
	LBE → OI → JPO (partial mediation)	0.171**	0.001	0.090	0.285

N = 310; * < 0.05, ** < 0.01, *** < 0.001; LBE, Leading By Example; OI, Organizational Identification; OPO, Organizational Psychological Ownership; JPO, Job Psychological Ownership.

CI^*^: This 95% confidence interval excludes zero; therefore the mediating effect is statistically significant at ρ < 0.01.

Leading by example had a significant positive effect on organizational psychological ownership (β = 0.332, ρ < 0.001). H1a is supported. Leading by example had a significant positive effect on job psychological ownership (β = 0.159, ρ < 0.05). H1b is supported. Leading by example had a significant positive effect on organizational identification (β = 0.506, ρ < 0.001). H2 is supported. Organizational identification had a significant positive effect on organizational psychological ownership (β = 0.608, ρ < 0.001). H3a is supported. Organizational identification had a significant positive effect on job psychological ownership (β = 0.341, ρ < 0.001). H3b is supported.

The estimated mediating effect of standardization in LBE → OI → OPO model is 0.286 (ρ < 0.001), and the 95% confidence interval was [0.199, 0.410], excluding 0. The mediating effect of this path is significantly supported. The direct effects of LBE → OI (β= 0.508, ρ < 0.001), and OI → OPO (β= 0.563, ρ < 0.001) pathways were significant, but the direct effect value of LBE → OPO was not significant **(β =** **0.147**, **ρ> 0.05)**, and the 95% confidence interval was **[−0.008, 0.310]**, including 0. Therefore, H4a is validated and fully mediated. Similarly, H4b is validated and partially mediated.

Finally, Tables 4, 5 present a summary of the estimated results, showing that H1a, H1b, H2, H3a, H3b, H4a, and H4b are supported. Among them, H4a is fully mediators and H4b is partially mediators.

## Analysis of Regulatory Effect of LMX

Using the SPSS 25.0 tool, hierarchical regression analysis was used to test the moderating effect of LMX on leading by example and organizational identification, organizational psychological ownership, and job psychological ownership. As shown in Table 6, ΔR^2^ values are 0.013, and showed significant changes (Sig. F change = 0.012), 0.011 (Sig. F change = 0.041), and 0.013 (Sig. F change = 0.017), respectively. Significant values tested by ANOVA are all 0.000 and VIF = 1.749, indicating that there is no serious collinearity problem.

**Table 6:** Summary of the regulatory effect of LMX.

**Variable**	**Dependent variable**											
	**OI**				**OPO**				**JPO**			
	**M1**	**M2**	**M3**	**M4**	**M5**	**M6**	**M7**	**M8**	**M9**	**M10**	**M11**	**M12**
Constant	6.033***	6.033***	6.033***	5.969***	5.531***	5.531***	5.531***	5.461***	5.594***	5.594***	5.594***	5.520***
Age	0.044	0.078	0.067	0.077	0.070	0.101	0.089	0.100	−0.063	−0.025	−0.038	−0.026
Marriage	0.059	0.027	−0.001	0.010	0.114	0.085	0.054	0.066	0.059	0.024	−0.007	0.006
degree of education	−0.030	−0.023	0.002	−0.009	0.016	0.022	0.050	0.038	0.017	0.024	0.052	0.039
Position grade	0.206**	0.167**	0.123*	0.131**	0.156*	0.121+	0.072	0.081	0.176*	0.133*	0.084+	0.093
LBE		0.456***	0.127*	0.190**		0.418***	0.054	0.123		0.508***	0.144	0.215*
LMX			0.453***	0.490***			0.502***	0.543***			0.502***	0.544***
LBE*LMX				0.136*				0.149*				0.155*
R^2^	0.046	0.263	0.361	0.375	0.031	0.153	0.234	0.244	0.029	0.229	0.318	0.331
ΔR^2^		0.217	0.098	0.013		0.122	0.080	0.011		0.200	0.089	0.013
F^2^	3.694**	21.728***	28.590***	25.853***	2.443*	10.998***	15.396***	13.941***	2.311	18.052***	23.557***	21.324***
Sig. F change	0.006	0.000	0.000	0.012	0.047	0.000	0.000	0.041	0.058	0.000	0.000	0.017
VIF				1.747				1.747				1.747
Durbin-Watson				1.757				1.858				1.684
N	310											

+ ρ < 0.1; *ρ < 0.05; **ρ < 0.01; ***ρ < 0.001.

**Figure 3:**
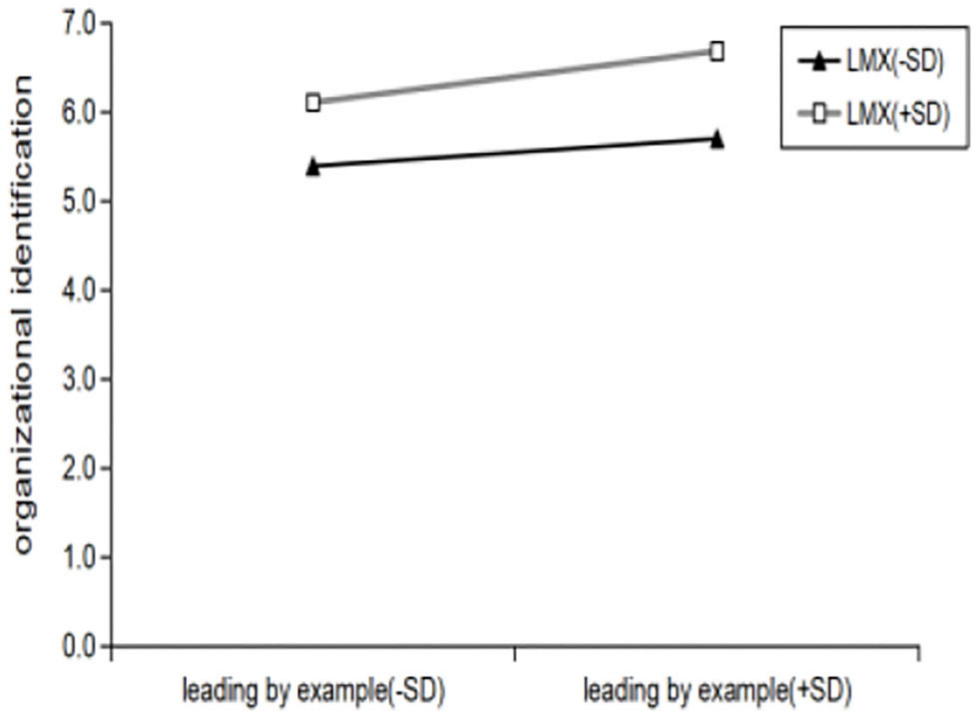
Interaction between LMX and leading by example in predicting organizational identification.

**Figure 4:**
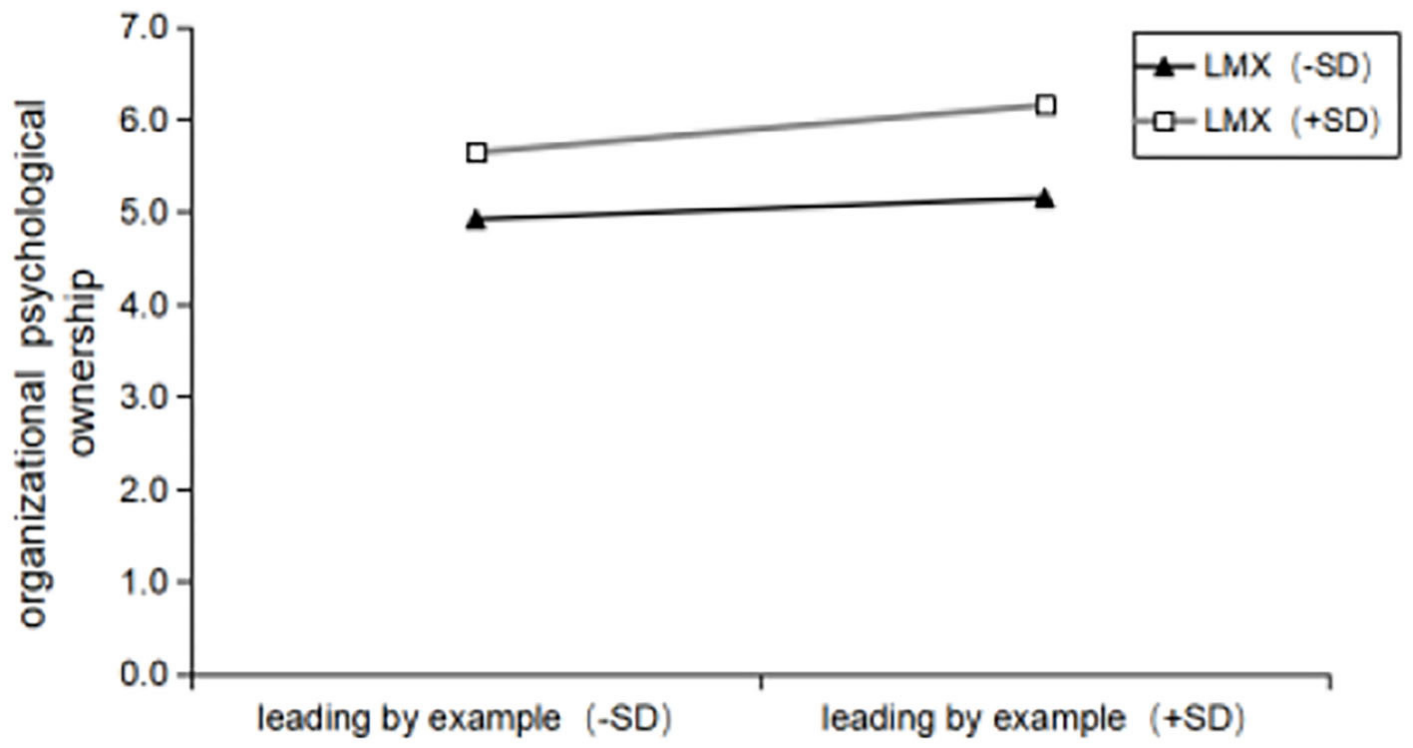
Interaction between LMX and leading by example in predicting organizational psychological ownership.

**Figure 5:**
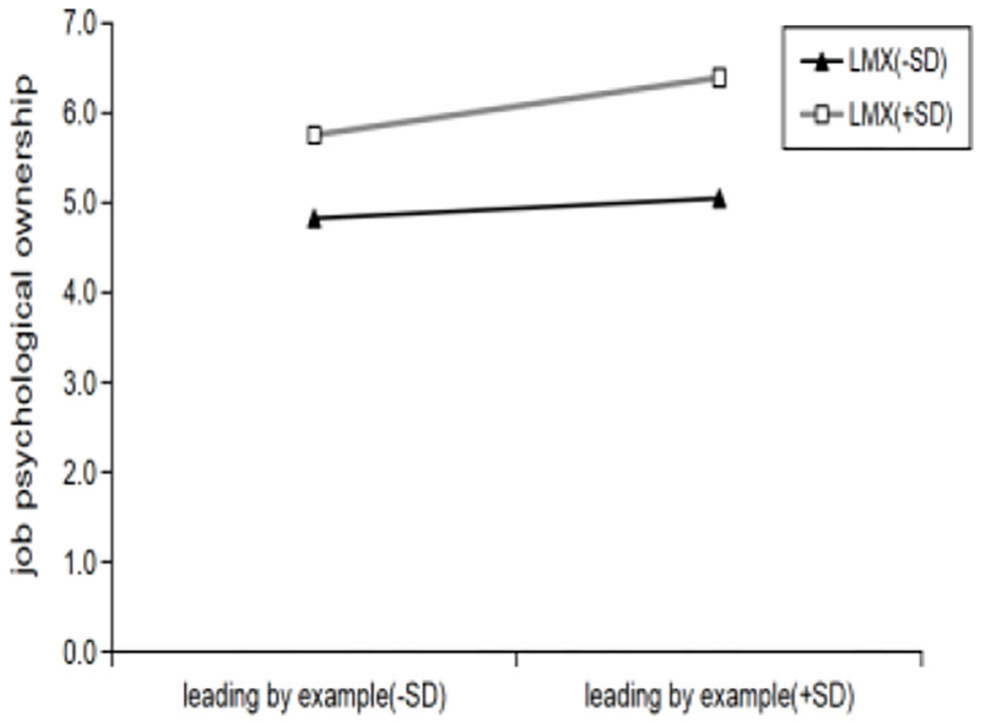
Interaction between LMX and leading by example in predicting job psychological ownership.

As confirmed above, leading by example had a significant positive effect on organizational identification (β = 0.506, ρ < 0.001). By referring to the method of Aiken and West (1991) to test the moderating effect, Simple Slope K was used to analyze the moderating effect of LMX. Introduced in the **Model (OI)** leading by example and LMX and interaction of the two items, the explanatory power of the model increases as (ΔR^2^ = 0.012), and leading by example and LMX of interaction has a significant negative effect on organizational identification **(β** **=** **0.136**, **ρ** **< 0.05)**. That is, the LMX has a significant positive moderating effect on the relationship between leading by example and organizational identification, its regulating effect is shown in Figure 3. In other words, compared with low LMX (k = 0.3471, R^2^ = 0.1612), when LMX is high (k = 0.2903, R^2^ = 0.0469), the relationship between leading by example and organizational identification is enhanced. Therefore, **hypothesis H6 is verified**. In a similar way, introduced in the **Model (OPO)** leading by example and LMX of interaction has a significant effect on organizational psychological ownership **(β** **=** **0.149**, **ρ** **< 0.05)**. That is,LMX has a significant positive moderating effect on the relationship between leading by example and organizational psychological ownership, its regulating effect is shown in Figure 4. Compared with low LMX (k = 0.24, R^2^ = 0.0668), when LMX is high (k = 0.0902, R^2^ = 0.0249), the relationship between leading by example and organizational psychological ownership is enhanced. **Hypothesis H5a is verified**. Introduced in the **Model (JPO)** leading by example and LMX of interaction has a significant negative effect on job psychological ownership **(β** **=** **0.155**, **ρ** **< 0.05)**. That is,the LMX has significant positive moderating effect on the relationship between leading by example and job psychological ownership, its regulating effect is shown in Figure 5. Compared with low LMX (k = 0.2731, R^2^ = 0.0827), when LMX is high (k = 0.4361, R^2^ = 0.0804), the relationship between leading by example and job psychological ownership is enhanced. **Hypothesis H5b is verified**.

In conclusion, the surprising thing is that all the hypotheses hold up.

## Discussion

This study verifies that leading by example has a significant positive impact on organizational psychological ownership and job psychological ownership. It is arguable in the Chinese context that employees believe that they can work with leaders who are presentable and lead by example. Hence, employees will increase their initiative in work and sense of ownership of the organization. Furthermore, leading by example behavior should be actively carried out in enterprises with increasingly flat organizations, which is helpful to perfect the authorized leadership system and enrich the empowering leadership theory.

**First**, we focused on the direct relationship of leading by example on employees' psychological ownership (organizational psychological ownership and job psychological ownership). The results support our research hypotheses H1a and H1b. Leading by example has a high level of commitment, responsibility, and dedication to their own work and that of their subordinates, and often play a good exemplary role, which will directly affect employees' psychological ownership of their job or organization (Lin and Ling, 2016). We believe that to a certain extent when employees believe that they can work with Leaders who are presentable and lead by example, employees will increase their initiative in work and sense of ownership of the organization.

**Second**, this study depicts organizational identification as mediating between leading by example and employees' organizational and job psychological ownership. The results support our research hypotheses H4a and H4b. If employees identify with the organization, they may perceive their work as more meaningful because their work supports their self-concept (Lee et al., 2018). When people produce identity to a group, will produce personalization, and groups have common feelings such as a feeling of fate, and there will be in-group favoritism, specific performance for organization members will be more willing to work with organizations more closely, more cooperative behavior, and organizational competition of more power and more organizational citizenship behavior (Tajfel and Turner, 1979). From an assumed perspective of the influence of leading by example on employees' psychological ownership, the results show that the interviewees' exemplary behavior of their leader or supervisor can indirectly affect employees' job psychological ownership and organizational psychological ownership through organizational identification. Therefore, enterprises should attach great importance to cultivating employees' organizational identification when implementing the authorized leadership system. Compared with employees' job psychological ownership, organizational identification plays a greater role in leading by example and employees' organizational psychological ownership. The perception of higher leading by example can stimulate higher organizational identification, thus promoting more expressive behavior of employees.

**Third**, this study examined the moderating effect of LMX on the direct relationship between leading by example and organizational identification and employees' psychological ownership (organizational psychological ownership and job psychological ownership). The results support our research hypotheses H5a, H5b, and H6. Leader–member exchange (LMX) itself is an interactive exchange process, in which the superior controls the allocation of resources and is in a favorable position of exchange, while the subordinate is in a disadvantageous position of passive acceptance. From the perspective of leaders, it is an important responsibility of them to mobilize the work enthusiasm of each employee to guide and motivate subordinates to strive to achieve individual and organizational performance goals. To increase members' involvement in work, leaders may take the initiative to repair and maintain the relationship with members and increase members' organizational identification, Organizational psychological ownership, and job psychological ownership, forming high-quality leader–member exchange. From the perspective of members, in order to meet the needs of personal development and self-realization, they also need to establish and maintain a good “relationship” with leaders, to stay in the “circle” and get more resources. Individuals' high commitment to work is an important weight for them to gain recognition and reward from superiors, and also helps them maintain high-quality exchange relationship with leaders.

## Practical Implications, Limitations, and Future Research

### Practical Implications

The results point out some practical significance for managers to establish a new model of leadership mechanism. **First**, as one of the important dimensions of empowering leadership, leading by example is helpful to improve the theory of empowering leadership. Leading by example should be actively carried out in enterprises with increasingly flat organizations, which is helpful to perfect the authorized leadership system and enrich the empowering leadership theory. **Second**, in this study, we found that organizational identification is an important mediating mechanism between leading by example and psychological ownership, revealing the implicit role and mechanism of organizational identification in their relationship, and enriching the connotation of how empowering leadership influences employees' psychological ownership. This study uses SEM to provide a new insight into the mediating mechanism behind these effects and provides a new path and theoretical framework for improving employees' intrinsic motivation and management performance. **Third**, under the influence of Chinese pan-family culture, the influence mechanism of leading by example on organizational identification, organizational psychological ownership, and job psychological ownership is differentiated by a different quality of LMX organization circle culture.

### Limitations and Future Research

This study is limited to enterprises and employees in China's labor-intensive property management industry. In the future, this model can be tested in other labor-intensive industries to observe possible differences, such as the construction industry and logistics industry.

Because organizational identification emphasizes self-identity, whereas organizational psychological ownership emphasizes the sense of ownership and control, the possible causal relationship between organizational psychological ownership and organizational identification can be discussed in the future. The causal effect of organizational psychological ownership on organizational identification under certain conditions can be discussed in the future. A high organizational psychological ownership can promote high organizational identification. It is important to avoid negative behavior with high organizational identification. As employees with high organizational identification pay too much attention to their responsibilities or behaviors beneficial to the organization when safeguarding the interests of the organization (Albert et al., 2000) while selectively ignoring unethical behaviors and even daring to challenge the negative effects of illegal behaviors (Umphress et al., 2010; Chen and Zhang, 2016).

Due to the influence of excessive trust on “insider in-group” in leader–member exchange, it will lead to the negative influence of excessive delegation of leading by example. The negative effects of excessive leader–member exchange will bring two aspects of “internal troubles” and “external troubles” to employees (Jiang and Xu, 2020). When employees perform behaviors to safeguard the interests of the organization, they will not consider the morality of the behavior itself too much. In this case, employees will pay more attention to their responsibility for the behavior or the possible beneficial impact of the behavior on the organization, and selectively ignore the moral meaning of immoral behavior (e.g., pro-organizational non-ethical behavior) (Rasool Samma et al., 2020).

The more tasks authorized, the greater the pressure of the authorized person, the longer the authorization time, the heavy work burden of the authorized person, easily lead to more obvious fatigue of the employee, thus affecting the work efficiency of the employee, resulting in negative impact.

In the future, a single dimension, such as Affect, Loyalty, Contribution, and Professional Respect, can be extracted from the four dimensions of LMX to test the regulatory effect.

## Conclusions

This research model of the independent variable is extracted from empowering leadership on sub-dimension demonstration (i.e., leading by example), and on this basis to build a developing country such as China still belongs to one of the new research paradigm. In this study, our findings support the link between leading by example, LMX, organizational identification, organizational psychological ownership, and job psychological ownership. The results of this study show that leading by example has a significant positive impact on organizational identification, organizational psychological ownership, and job psychological ownership. This study also verified the mediating effect of organizational identification on the relationship between leading by example and employee's organizational and job psychological ownership, and verified that LMX has a significant moderating effect on the relationship between leading by example and organizational identification, and employee's organizational and job psychological ownership.

**First**, this study verifies that leading by example has a significant positive impact on organizational psychological ownership and job psychological ownership. Such conclusion and Li et al. (2018) and Jiang et al. (2019) the research results of similar, they confirmed that the empowering leadership through measures such as leading by example, highlights the meaningfulness of work (Ahearne et al., 2005), participation in decision-making (Zhang and Bartol, 2010), provides employees with a great degree of autonomy (Ahearne et al., 2005), make the employee's individual career development and work ability get promoted, so as to improve employees' work and the sense of belonging to an organization, gradually formed to organizational and job psychological ownership. Hence, strengthening leading by example will help to enhance employees' enthusiasm for the job and sense of ownership of the organization (Wu et al., 2021).

**Second**, mediating mechanism of organizational identification; the results show that organizational identification plays a mediating role between leading by example and employee's organizational and job psychological ownership. Among them, organizational identification plays a completely mediating role in the influence of leading by example on organizational psychological ownership; organizational identification plays a partial mediating role in leading by example and job psychological ownership. When leaders' exemplary behaviors in the organization are consistent with employees' inner values, employees can feel the consistency with the organization, have a strong sense of membership, and then show high loyalty in their behaviors (Hameed et al., 2022). They will take the organization as their “home” and are more willing to make contributions to maintain the values and goals of the organization, and even sacrifice personal interests, resulting in a high degree of organizational psychological ownership (Xu et al., 2022). Members with high organizational identification have consistency and emotional connection with the organization in concept and behavior, such as compliance with the rules and regulations of the enterprise. Therefore, positive organizational identification leads to higher organizational psychological ownership. They regard work as their own work, internalize corporate goals as personal goals, and naturally produce job psychological ownership in the process of realizing the personal value and maintaining organizational value (Helen et al., 2016; Hameed et al., 2022). Therefore, positive organizational identification leads to higher job psychological ownership. Hence, employees will increase their initiative in work and sense of ownership of the organization.

**Third**, we examined the moderating effects of LMX. The results show that LMX plays a moderating role in the relationship between leading by example and organizational identification, and organizational and job psychological ownership. Under the influence of pan-family culture, Chinese employees often expect their organizations to have families (Zhou et al., 2012; Martin et al., 2017). Both leaders and employees expect to become members of the circle, and they have a strong sense of attachment, loyalty, and control to the organization and work, showing a strong organizational identification, organizational psychological ownership, and job psychological ownership (Wu and Zhang, 2017; Omilion-Hodges and Ptacek, 2021). In other words, the influence mechanism of leading by example on organizational identification, organizational psychological ownership, and job psychological ownership varies with organizational Circle Culture, which also sets boundary conditions for the application of our theory. It fully demonstrates that there is a strong Circle Culture in the organizational system of Chinese enterprises.

## Data Availability Statement

The raw data supporting the conclusions of this article will be made available by the authors, without undue reservation.

## Ethics Statement

The studies involving human participants were reviewed and approved by Zhejiang Shuren University, Hangzhou Research Ethics Review Committee. The patients/participants provided their written informed consent to participate in this study.

## Author Contributions

ZY designed the research idea, developed the hypotheses, drafted the final manuscript, analyzed the results, and drafted the research methodology. XS and ZS supervised this research and suggested extensive revisions during the research work and participated in data collection and analysis. YX worked on the discussion part of the article. All authors read and approved the final manuscript.

## Funding

This article was supported by the Open Fund Project of Zhejiang Modern Services Research Center (SXFJZ202202).

## Conflict of Interest

The authors declare that the research was conducted in the absence of any commercial or financial relationships that could be construed as a potential conflict of interest.

## Publisher's Note

All claims expressed in this article are solely those of the authors and do not necessarily represent those of their affiliated organizations, or those of the publisher, the editors and the reviewers. Any product that may be evaluated in this article, or claim that may be made by its manufacturer, is not guaranteed or endorsed by the publisher.
