# Supplementary material for: Impact of Leading by Example on Employees' Organizational and Job Psychological Ownership: A Moderated Mediation Study
Source: Front Psychol. 2022 Jul 6;13:888653. doi: 10.3389/fpsyg.2022.888653 (PMC9298667; doi:10.3389/fpsyg.2022.888653)
Supplement: Supplementary file 1 [file Data_Sheet_1.docx]

**Appendix**

**Survey questionnaire**

Dear Sir/Madam：

Hello! Thank you for taking the time to answer this questionnaire. Your participation is my greatest support and help.

This questionnaire is for academic research purposes only and has nothing to do with job evaluation and confidential information. You only need to answer according to your true feelings, and our survey is anonymous, so you don't have to worry about personal information disclosure and impacts on the work and other issues. Please believe that we respect and protect the participants. It takes about 5 min to fill in the form. Again, thank you for your earnest reply.

Best wishes: smooth work and happy life!

Please mark the following questions with “**√** “or with an “○ “that most closely matches how you actually feel at work.

**Demographics**

| **Your name or initials(example: Pan**  **Hong Bing→PHB)** | **____ ____ ____** |
| --- | --- |
| **Gender/Marriage** | ① Male ( )  ② Female ( )  ③ Married ( )  ④ Unmarried ( ) |
| **Education** | ① Junior high school and below( )  ② Vocational High School Education ( )  ③ Junior College ( )  ④ Regular College Course ( )  ⑤ Master( )  ⑥ Doctor( ) |
| **Nationality/Religion** | ① Nationality ( )  ② Place of Birth（ ）  ③ Religion（ ） |
| **Year of Birth/Age** | Year of birth（ ）, age( ) |
| **Rank/Position** | ① Employee ( )  ② Lower management ( )  ③ Pro-manager ( )  ④ Manager ( )  ⑤ Top management ( )  ⑥ Else ( ) |
| **Years of Working** | Years of Working（ ） |

**Questionnaire**

| **Leading By Example (LBE,1-6)** | | | | | | | | |
| --- | --- | --- | --- | --- | --- | --- | --- | --- |
| **BLE.1** | Sets high standards for performance by his/her own behavior | 1 | 2 | 3 | 4 | 5 | 6 | 7 |
| **BLE.2** | Works as hard as he/she can | 1 | 2 | 3 | 4 | 5 | 6 | 7 |
| **BLE.3** | Works as hard as anyone in my work group | 1 | 2 | 3 | 4 | 5 | 6 | 7 |
| **BLE.4** | Sets a good example by the way he/she behaves | 1 | 2 | 3 | 4 | 5 | 6 | 7 |
| **BLE.5** | Leads by example | 1 | 2 | 3 | 4 | 5 | 6 | 7 |
| **Organizational Identification Scale (OI, 1-6)** | | | | | | | | |
| **OI.1** | When someone criticizes my organization, it  feels like a personal insult | 1 | 2 | 3 | 4 | 5 | 6 | 7 |
| **OI.2** | I am very interested in what others think  about my organization | 1 | 2 | 3 | 4 | 5 | 6 | 7 |
| **OI.3** | When I talk about this organization, I  usually say ' we' rather than 'they' | 1 | 2 | 3 | 4 | 5 | 6 | 7 |
| **OI.4** | This organization's successes are my successes | 1 | 2 | 3 | 4 | 5 | 6 | 7 |
| **OI.5** | When someone praises this organization, it  feels like a personal compliment | 1 | 2 | 3 | 4 | 5 | 6 | 7 |
| **OI.6** | If a story in the media criticized the  organization, I would feel embarrassed | 1 | 2 | 3 | 4 | 5 | 6 | 7 |
| **Psychological Ownership Scale (PO, 1-12)**  **Organization-based Psychological Ownership (OPO, PO 1-6)**  **Job-based Psychological Ownership (JPO, PO 7-12)** | | | | | | | | |
| **OPO.1** | This is MY organization | 1 | 2 | 3 | 4 | 5 | 6 | 7 |
| **OPO.2** | I sense that this organization is OUR company | 1 | 2 | 3 | 4 | 5 | 6 | 7 |
| **OPO.3** | I feel a very high degree of personal ownership for this organization | 1 | 2 | 3 | 4 | 5 | 6 | 7 |
| **OPO.4** | I sense that this is MY company | 1 | 2 | 3 | 4 | 5 | 6 | 7 |
| **OPO.5** | This is OUR company | 1 | 2 | 3 | 4 | 5 | 6 | 7 |
| **OPO.6** | Most of the people that work for this  organization feel as though they own the  company | 1 | 2 | 3 | 4 | 5 | 6 | 7 |
| **JPO.1** | This is MY job | 1 | 2 | 3 | 4 | 5 | 6 | 7 |
| **JPO.2** | I feel a very high degree of personal  ownership for this job | 1 | 2 | 3 | 4 | 5 | 6 | 7 |
| **JPO.3** | I sense that this is MY job | 1 | 2 | 3 | 4 | 5 | 6 | 7 |
| **JPO.4** | Most people that work for this organization  feel as though they own their job | 1 | 2 | 3 | 4 | 5 | 6 | 7 |
| **JPO.5** | It is hard for me to think about this job as  MINE (reversed) | 1 | 2 | 3 | 4 | 5 | 6 | 7 |
| **JPO.6** | I think that I could easily become as  attached to another organization as I am to  this one (reversed) | 1 | 2 | 3 | 4 | 5 | 6 | 7 |
| **Leader-Member Exchange(LMX, 1-11)** | | | | | | | | |
| LMX.1 | Like my supervisor very much as a person. | 1 | 2 | 3 | 4 | 5 | 6 | 7 |
| LMX.2 | My supervisor is the kind of person one  would like to have as a friend | 1 | 2 | 3 | 4 | 5 | 6 | 7 |
| LMX.3 | My supervisor is a lot of fun to work with | 1 | 2 | 3 | 4 | 5 | 6 | 7 |
| LMX.4 | My supervisor defends my work actions to a  superior, even without complete knowledge  of the issue in question | 1 | 2 | 3 | 4 | 5 | 6 | 7 |
| LMX.5 | My supervisor would come to my defense if  I were "attacked" by others | 1 | 2 | 3 | 4 | 5 | 6 | 7 |
| LMX.6 | My supervisor would defend me to others in  the organization if I made an honest mistake | 1 | 2 | 3 | 4 | 5 | 6 | 7 |
| LMX.7 | Do work for my supervisor that goes beyond  what is specified in my job description | 1 | 2 | 3 | 4 | 5 | 6 | 7 |
| LMX.8 | I am willing to apply extra efforts, beyond  those normally required, to further the  interests of my work group | 1 | 2 | 3 | 4 | 5 | 6 | 7 |
| LMX.9 | I am impressed with my supervisor's  knowledge of his/her job | 1 | 2 | 3 | 4 | 5 | 6 | 7 |
| LMX.10 | I respect my supervisor's knowledge of and  competence on the job | 1 | 2 | 3 | 4 | 5 | 6 | 7 |
| LMX.11 | I admire my supervisor's professional skills | 1 | 2 | 3 | 4 | 5 | 6 | 7 |
